# Zein nanoparticles as nontoxic delivery system for maytansine in the treatment of non-small cell lung cancer

**DOI:** 10.1080/10717544.2019.1704942

**Published:** 2019-12-24

**Authors:** Xianglong Yu, Huichao Wu, Haiyan Hu, Ziyi Dong, Yunni Dang, Qi Qi, Yan Wang, Shouying Du, Yang Lu

**Affiliations:** School of Chinese Materia Medica, Beijing University of Chinese Medicine, Beijing, China

**Keywords:** Maytansine, Zein, lung cancer, nanoparticle, A549

## Abstract

*Purpose:* Maytansine (DM1) is a potent anticancer drug and limited in clinical application due to its poor water solubility and toxic side effects. Zein is widely used in nano drug delivery systems due to its good biocompatibility. In this study, we prepared DM1-loaded zein nanoparticles (ZNPs) to achieve tumor targeting and reduce toxic side effects of DM1. *Methods:* ZNPs were prepared by phase separation and Box-Behnken design was used to optimize the formulation. Then, confocal fluorescence microscope and flow cytometry were used to determine cellular uptake of ZNPs. A549 cells were cultured *in vitro* to study cytotoxicity and used to establish tumor xenografts in nude mice. Biodistribution and antitumor activity of ZNPs were performed *in vivo* experiments. In addition, we also performed histological and immunohistochemical examinations on tumors and viscera. *Results:* The optimal prescription was obtained by using 120** **μL zein added to 2** **mL water under stirring in 300** **rpm. The encapsulation efficiency and drug loading were 82.97** **±** **0.80% and 3.32** **±** **0.03%, respectively. We found that DM1-loaded ZNPs have a strong inhibitory effect on A549 cells, which stemmed from the ability of ZNPs to enhance cellular uptake. Furthermore, we demonstrated that DM1-loaded ZNPs exhibits a better antitumor efficacy than DM1, which tumor inhibition rate were 97.3% and 92.7%, respectively. The biodistribution revealed that ZNPs could targeted to tumor. Finally, we confirmed by histological that DM1-loaded ZNPs are nontoxic. *Conclusion:* DM1-loaded ZNPs have considerable antitumor activity. Thus, DM1-loaded ZNPs are a promising treatment of non-small cell lung cancer.

## Introduction

Lung cancer remains the leading cause of cancer-related mortality worldwide and non-small cell lung carcinoma (NSCLC) represents approximately 85% of all new lung cancer diagnosis (Fitzmaurice et al., 2018; Prabhu et al., 2018). Maytansine (DM1) is a powerful tubulin polymerization inhibitor whose antitumor mechanism inhibits cell mitosis like vinblastine and vincristine, but its antitumor activity *in vitro* was higher than vincristine and paclitaxel 20–100 times, 24–270 times, respectively (Issell & Crooke, 1978; Wishart et al., 2008). Therefore, DM1 can effectively treat various malignancies including breast cancer, melanoma, multiple myeloma, liver cancer and lung cancer (Kusari et al., 2016; Zhong et al., 2017). Although DM1 has high antitumor activity, its clinical application was limited due to strong side effects, narrow therapeutic window and poor water solubility (Kupchan et al., 1972; Blum et al., 1978; Junttila et al., 2011). These properties make it promising as a targeted drug. In order to overcome those effects of DM1 and improve clinical application, antibody-drug conjugates (AMCs) are currently the most widely used technology. At present, more than ten types of antibody-maytansinoid conjugates have entered various phases of clinical trials (Chudasama et al., 2016; de Goeij & Lambert, 2016; Taplin et al., 2018). It has to be noted, however, that the clinical use of AMCs, is challenged by their poor stability, low drug content, high cost, small scale production, relatively narrow therapeutic index, limited clinical success, off-target toxicities of payloads and potential immunogenicity (Perez et al., 2014; Tolcher, 2016; Mecklenburg, 2018).

Nanodrug delivery systems are capable of prolonging blood circulation time, numerous renewable sources, high drug-binding, improving drug solubilization, and accumulating at a tumor via the enhanced permeability and retention (EPR) effect (Elzoghby et al., 2017; Pang et al., 2018). Zein, for this purpose, could be a good carrier in this system due to its inherent biocompatibility, nontoxicity, *in vivo* biodegradability and the capacity of self-assembly (Chen et al., 2019). It is classified as one of the safest biomaterial excipients by the US FDA (Labib, 2018). Moreover, compared with other proteins, zein has larger proportion of hydrophobic amino acid, which leads to higher potential for hydrophobic drug loading and self-assembling into stable nanoparticles without the use of toxic chemical crosslinkers (Labib, 2018; Pang et al., 2018).

In this study, DM1-loaded ZNPs were prepared by phase separation method and assessed as a systemic drug delivery vehicle in treatment of lung cancer. The microstructure of the nanoparticles and anti-proliferative effects on A549 cells were studied, *in vitro* cellular uptake and the biodistribution were investigated in detail. The platform improved drug delivery to the tumor and produced significant efficacy. The ZNPs drug carrier could prove useful in the treatment of lung cancer and is worthy of further pre-clinical investigation in the oncology setting.

## Material and methods

### Materials

Zein (Meilun Biological, Dalian, China). N2'-deacetyl-N2'-(3-mercapto-1-oxopropyl)-maytansine (DM1 > 98%, Bright Gene Co., Ltd., Suzhou, China). Dimethyl sulfoxide (DMSO, Bailunsi, Tianjin, China). 2-[2-[2-Chloro-3-[(1,3-dihydro-3,3-dimethyl-1-propyl-2H-indol-2-ylidene) ethylidene]-1-cyclohexen-1-YL] ethenyl]-3,3-dimethyl-1-propylindolium iodide (IR-780 iodide, Alfa Aesar, Tianjin, China). Hoechst (Beyotime Biotechnology, Shanghai, China). Cell Counting Kit-8 (CCK-8, Dojindo, Shanghai, China). Dulbecco's modified eagle's medium (DMEM, Solarbio, Beijing, China). Fetal bovine serum (FBS, Gibco, Grand Island, NY). Trypsin-EDTA (Gibco). All materials were used without further purification.

### Preparation of ZNPs

DM1-loaded ZNPs were prepared by phase separation method. The DM1 and zein were formulated into a solution at a concentration of 5** **mg/mL and 60** **mg/mL, respectively (DMSO dissolved). The 60** **µL of DM1 solution and certain volume of zein solution were mixed and the mixture was slowly dropped into a certain volume of distilled water with stirring. When the mixture is completely added to the water, the stirring will be terminated and obtained the DM1-loaded ZNPs.

### Drug encapsulation and loading efficiency

For evaluation of drug entrapping and loading efficiency, the prepared DM1-loaded ZNPs were centrifuged at 10,000** **rpm for 55** **min to remove the free DM1. Then the free DM1 was diluted with methanol and the concentration of DM1 was evaluated using high performance liquid chromatography (SHIMADZU, LC-20AD, Japan) at 245** **nm. Drug encapsulation efficiency and loading were determined by following equations respectively. Drug encapsulation efficiency = mass of drug on ZNPs/mass of feed drug × 100. Drug loading efficiency = mass of drug on ZNPs/mass of ZPNs × 100.

### Optimization of the formulation

The optimization was applied to determine the encapsulated efficiency of the drug. The Box-Behnken design was used (Table 1). The three factors are the volume of the zein solutions (*X*_1_), stirring speed (*X*_2_), water volume (*X*_3_).

**Table 1.** The levels and factors of Box-Behnken design.

Levels	Factors		
	*X*_1_ (µL)	*X*_2_ (rpm)	*X*_3_ (mL)
1	120	200	1
2	160	500	2
3	200	800	3

The three factors were the volume of the zein solutions (*X*_1_), stirring speed (*X*_2_), water volume (*X*_3_).

### Characterization of the ZNPs

The morphology and structure of the ZNPs was observed by field transmission electron microscopy (TEM) (JEM-2100, Japan). Briefly A drop of diluted ZNPs was placed on a 400 mesh carbon-coated copper grid. After drying, the samples were dyed using 2% sodium phosphotungstate. The size, distribution and zeta potential of the ZNPs were measured by dynamic light scattering spectrometer (Malvern, Nano-ZS90, UK). The ZNPs was analyzed after diluted with deionized water to a favorable concentration required for DLS.

### *In vitro* release

DM1 release profile assay was determined by measuring the residual amount of DM1 present in NPs (Rong et al., 2018; Sally et al., 2018). Briefly, 100** **µL of DM1-loaded ZNPs were redisposed in 900** **µL of distilled water containing 0.2% (w/v) Tween 80 and were shaken for 100** **rpm at 37** **°C. At certain intervals (0, 1, 2, 4, 6, 8, and 24** **h), the sample was centrifuged at 3000** **rpm for 5** **min, take 200** **µL of the supernatant, add 200** **µL of methanol, vortex for 30** **s, sonicated for 1** **min, and the amount of DM1 released was analyzed by HPLC. All samples were run in triplicates.

### Stability of DM1-loaded ZNPs

The storage stability of DM1-loaded ZNPs were evaluated by the change of particle size and drug leakage in distilled water at 4** **°C for 48** **h. At prearranged time (0, 1, 2, 6, 12, 24, 36, and 48** **h), samples were withdrawn and determined. The plasma stability of above ZNPs were also monitored by incubation the samples with FBS (1:9, v:v) and kept at room temperature. At prearranged time (0, 2, 6, 12, and 24** **h), samples were collected and measured (Lei et al., 2019).

### Cell culture

A549 cells (Human lung cancer) were obtained from the China Military Medical Science Academy of the PLA (Beijing, China). The cells were cultured in DMEM supplemented with 10% (v/v) FBS and incubated in a humidified incubator at 37** **°C with 5% (v/v) CO2.

### *In vitro* cell viability assay

To study the cell viability of DM1-loaded ZNPs. A549 cells were incubated in 96-well plate with 5** **×** **10^3^ cells per well in 100** **µL of complete medium for overnight until adherent and the cell monolayer coverage is up to 80%. Then cells were incubated with various concentrations of free DM1, DM1-loaded ZNPs and blank ZNPs (without of DM1) for 48** **h. The standard cell counting kit-8 (cck-8) assay was carried out to determine the cell viabilities relative to control untreated cells.

### Cellular uptake study

For study the cellular uptake of DM1-loaded ZNPs, we prepared IR-780-loaded ZNPs. A549 cells were seeded at a density of 4** **×** **10^5^ cells per well in 6-well plate, incubated for 24** **h. A549 cells were incubated with IR-780-loaded ZNPs with a IR-780 concentration in 2** **µg/mL at 37** **°C for 2, 4, 8** **h. Then A549 cells were washed two times with PBS solution and harvested by trypsin–EDTA digestion following centrifugation at 1000** **rpm for 3** **min. The cells were re-suspended in 200** **µL of PBS (pH 7.4) and analyzed using a flow cytometer (Beckman Coulter, MoFlo XDP, US), where 10,000 cells were recorded for each sample.

### Confocal laser scanning microscopy

A549 cells were seeded at a density of 4** **×** **10^5^ cells per dish in 20** **mm culture dish (NEST, Wuxi, China) and treated with IR-780-loaded ZNPs with a IR-780 concentration in 2** **µg/mL at 37** **°C for 2, 4, 8** **h. After washing with PBS (pH** **=** **7.4) for three times, the cell nuclei were labeled with Hoechst and then imaged by the confocal fluorescence microscope (Olympus, FV 1000, Japan) with a 60× oil objective.

### Animal model

Female nude mice were purchased from SPF (Beijing) Biotechnology Co., Ltd. and Animal care was performed in compliance with the guidelines of the Ministry of Science and Technology of China (2006) and the related ethical regulations of Beijing University of Chinese Medicine. To develop the tumor model, 3** **×** **10^6^ A549 cells suspended in 200** **μL complete medium were injected subcutaneously on each flank of mice. The mice were used when tumor volumes reached about 100–250 mm^3^.

### *In vivo* imaging

For study biodistribution in tumor-bearing mice using an *in vivo* imaging system MetaMorph-MIIS (Molecular Devices, CA). 200** **µL IR-780-loaded ZNPs or free IR-780 with 100** **µg/mL IR-780 equivalent concentration was intravenously (i.v.) injected into each mouse. The mice were anesthetized by intraperitoneal injection of 5% (w/v) chloral hydrate. To detect IR-780 fluorescence, we used 740** **nm as the excitation light, and collected emission spectra from 780** **nm to 850** **nm. Full body images were obtained at 2, 6, 24, 60, 72** **h after injection. The mice were sacrificed 60** **h after i.v. injection and major organs were harvested, including the tumor, liver, heart, lung, spleen, and kidneys for ex vivo imaging. Relative signal intensity in the organs was calculated, using Integrated Morphometry Analysis software (Molecular Devices, CA).

### Treatment efficacy

The antitumor treatment efficacy was investigated in A549 tumor-bearing mice. Nude mice bearing subcutaneous A549 tumors (100–150 mm^3^) were divided into 5 groups (*n*** **=** **4): (a) i.v. injected with 0.8** **mg DM1 equiv./kg DM1-loaded ZNPs; (b) i.v. injected with free DM1 0.8** **mg/kg; (c) i.v. injected with 0.8** **mg/kg ZNPs (without of DM1); (d) i.v. injected with PBS; (e) control (no treatment). The mice weights and tumor sizes were recorded every 2** **days for 15** **days, with their lengths and widths measured by a digital caliper. The tumor volume was calculated according to the following formula: width^2^ × length/2 (Labib, 2018; Prabhu et al., 2018). At day 15, the mice of each group were sacrificed and tumors were harvested and weighed. Tumor inhibition rate (TIR) was calculated according to the following formulas: (1 – (mean tumor weight of DM1 or zein treated group/mean tumor weight of control group)) ×100 (Zhong et al., 2017). Major organs were harvested and fixed in 4% paraformaldehyde for histological and immunohistochemical examinations.

### Statistics

Optimization of the formulation data was analyzed by design expert 10.0 and all other studies Statistical comparison was carried out according to the Mann-Whitney *U* test. *p* values** **<0.5 were considered statistically significant.

## Results

### Optimization of the formulation

The results of Box-Behnken design are shown in Table 2. The optimal formulation was obtained by using expert design 10.0 software: zein, 120** **µL; Stirring speed, 300** **rpm; water volume, 2** **mL. Estimated encapsulation efficiency was 81.64%, drug loading was 3.24%. In order to validate it, three parallel tests were performed using the optimized preparation conditions. The encapsulation efficiency and the drug loading were 82.97** **±** **0.80% and 3.32** **±** **0.03% respectively.

**Table 2.** The results of Box-Behnken design.

Formulation	Factors			Results	
	*X*_1_ (µL)	*X*_2_ (rpm)	*X*_3_ (mL)	Encapsulation efficiency (%)	Drug load (%)
1	120	200	2	82.20	3.29
2	200	500	1	71.39	1.74
3	120	500	1	78.89	3.16
4	160	500	2	81.37	2.47
5	160	200	3	77.57	2.35
6	160	500	2	78.63	2.38
7	120	500	3	73.58	2.94
8	200	800	2	82.71	2.02
9	200	500	3	77.94	1.90
10	160	500	2	80.54	2.44
11	160	800	1	77.27	2.34
12	160	500	2	78.79	2.39
13	160	800	3	77.67	2.35
14	160	200	1	78.30	2.37
15	160	500	2	81.78	2.48
16	200	200	2	80.45	1.96
17	120	800	2	81.83	3.27

The three factors were the volume of the zein solutions (*X*_1_), stirring speed (*X*_2_), water volume (*X*_3_).

### Characterization of the optimal nanoparticle formulation

**Figure 1.**
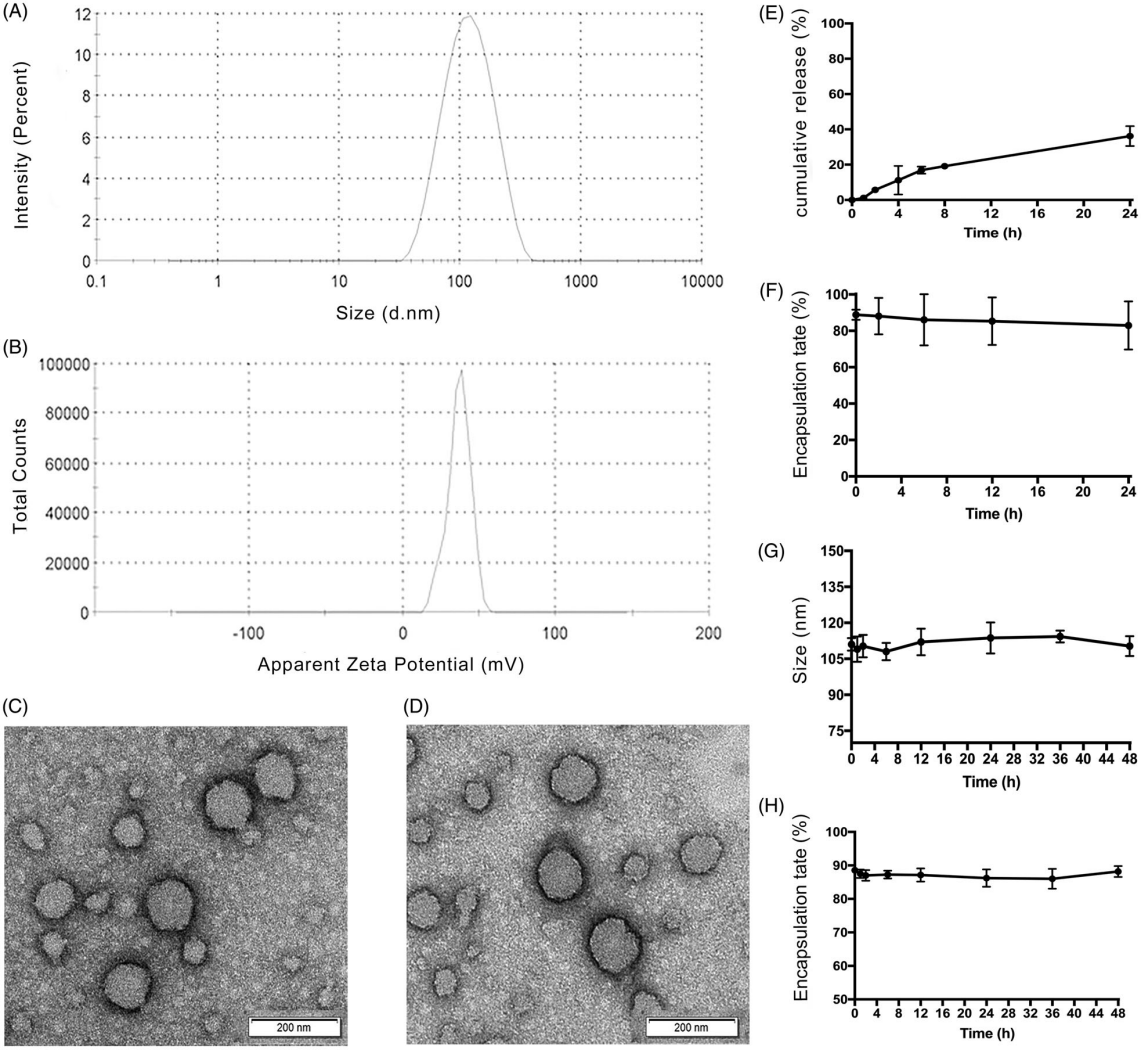
Characterization of ZNPs. (A) The size distributions of DM1-loaded ZNPs. (B) The zata potentials of DM1-loaded ZNPs. (C and D) The TEM image of DM1-loaded ZNPs. (C) DM1-loaded ZNPs. (D) ZNPs (without DM1). (E) In vitro release of DM1 from DM1-loaded ZNPs in distilled water containing 0.2% tween 80 at 100** **rpm and 37 °C. Encapsulation rate of DM1-loaded ZNPs after incubation with FBS solution for 24** **h at 37 °C (F). Store stability of DM1-loaded ZNPs showing their particle size change with time (G), and its corresponding encapsulation rate change with time (H) of DM1-loaded ZNPs.

Transmission electron microscopy showed that the DM1-loaded ZNPs (Figure 1(C)) and ZNPs (Figure 1(D)) had a smooth surface with a spherical shape, which indicated that DM1 could not change the size of ZNPs. The average diameter and the zeta potential for DM1-loaded ZNPs were 112.3** **±** **6.16** **nm and 37.0** **±** **1.14** **mV (*n*** **=** **3) respectively (Figure 1(A,B)). The polymer dispersity index (PDI) of DM1-loaded ZNPs was 0.213** **±** **0.02, which proves that the ZNPs has a uniform molecular weight distribution and the most particles were between 105.3 and 117.6** **nm. The presence of surface charge prevents particle aggregation, so the magnitude of zeta potential gives an indication of the potential stability of the colloidal system. The zeta potential of nanoparticles above ±30** **mV have been shown to be stable in suspension and the DM1-loaded ZNPs with zeta potentials 37.0** **±** **1.14** **mV are normally considered stable.

### *In vitro* release

In vitro release kinetics of DM1-loaded ZNPs was tested in distilled water containing 0.2% tween 80 medium at 37** **°C, as displayed in Figure 1(E). The results showed that the DM1 release from the ZNPs was biphasic characterized by initial fast release of about 20% of drug during the first 8** **h followed by a second phase of slow release with about 40% of DM1 was released after 24** **h. ZNPs are core-shell nanoparticles. In the preparation process, zein first forms a core, and then slowly adsorbs free zein to form shell a layer by layer (Li et al., 2017). So, the initial burst of drug may be due to some of the drug in the shell or at the core-shell interface, whereas the slow drug release phase could be assigned to the fraction of the drug physically entrapped within the hydrophobic core of the ZNPs (Sally et al., 2018).

### Stability of DM1-loaded ZNPs

The outstanding stability of DM1-loaded ZNPs is crucial to clinical applications, including storage stability *in vitro* and prolonged biological stability for drug targeting and circulation in vivo. The stability of ZNPs under physiological conditions was assessed using FBS. As shown in Figure 1(F), the results showed that only a small amount of DM1 leaked into the serum within 24** **hours, and no obvious adsorption precipitation was observed in the reaction solution, indicating that DM1-loaded ZNPs can remain stable in serum. It was well known that positively-charged nanoparticles are easily combined with negatively-charged red blood cell membranes, which can induce hemagglutination and hemolysis (Sally et al., 2018). We observed that positively-charged ZNPs remained stable in FBS. This may be because the nanocarrier has a large surface to volume ratio, which can interact with biomolecules such as proteins, nucleic acids and lipids in the blood. Since the protein is adsorbed on the nanocarrier surface leading to formation of nanoparticle-protein corona, which may be change ZNPs zeta potential and rapidly covered with opsonins and hence reducing the biological reactivity of NPs. The hydrophilic shell of ZNPs can reduce the adsorption of proteins as well as protection of the hydrophobic core from biological invasion (Sally et al., 2018; Saadat et al., 2019). Meanwhile, no significant change was observed in particle size and drug leakage within 48** **h for DM1-loaded ZNPs (Figure 1(G,H)), indicated that the ZNPs could remain good stability at 4** **°C for up to 48** **h.

### *In vitro* cytotoxicity

**Figure 2.**
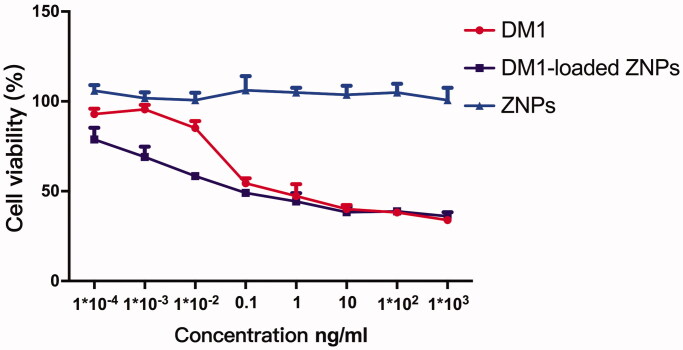
In vitro cell viability assay of A549 cells after the treatment with free DM1, DM1-loaded ZNPs and ZNPs (without DM1) for 48** **h.

The cytotoxicity of free DM1, DM1-loaded ZNPs and ZNPs (without DM1) was assessed with A549 cells by enzyme-labeled instrument (Thermo, MA) assay, as shown in Figure 2. It can be found that DM1 exerts dose-dependent anti-proliferation activity and ZNPs has no cytotoxicity. Interestingly, the half-maximal inhibitory concentration (IC50) values of free DM1 and DM1-loaded ZNPs were 0.04452** **ng/mL and 0.01237** **ng/mL, respectively. The result indicated that low dose DM1-loaded ZNPs exhibit stronger anti-proliferation capacity in A549 cells compare to free DM1, but the difference tends to be consistent after the dose reaches 0.1** **ng/mL.

### *In vitro* cellular uptake

**Figure 3.**
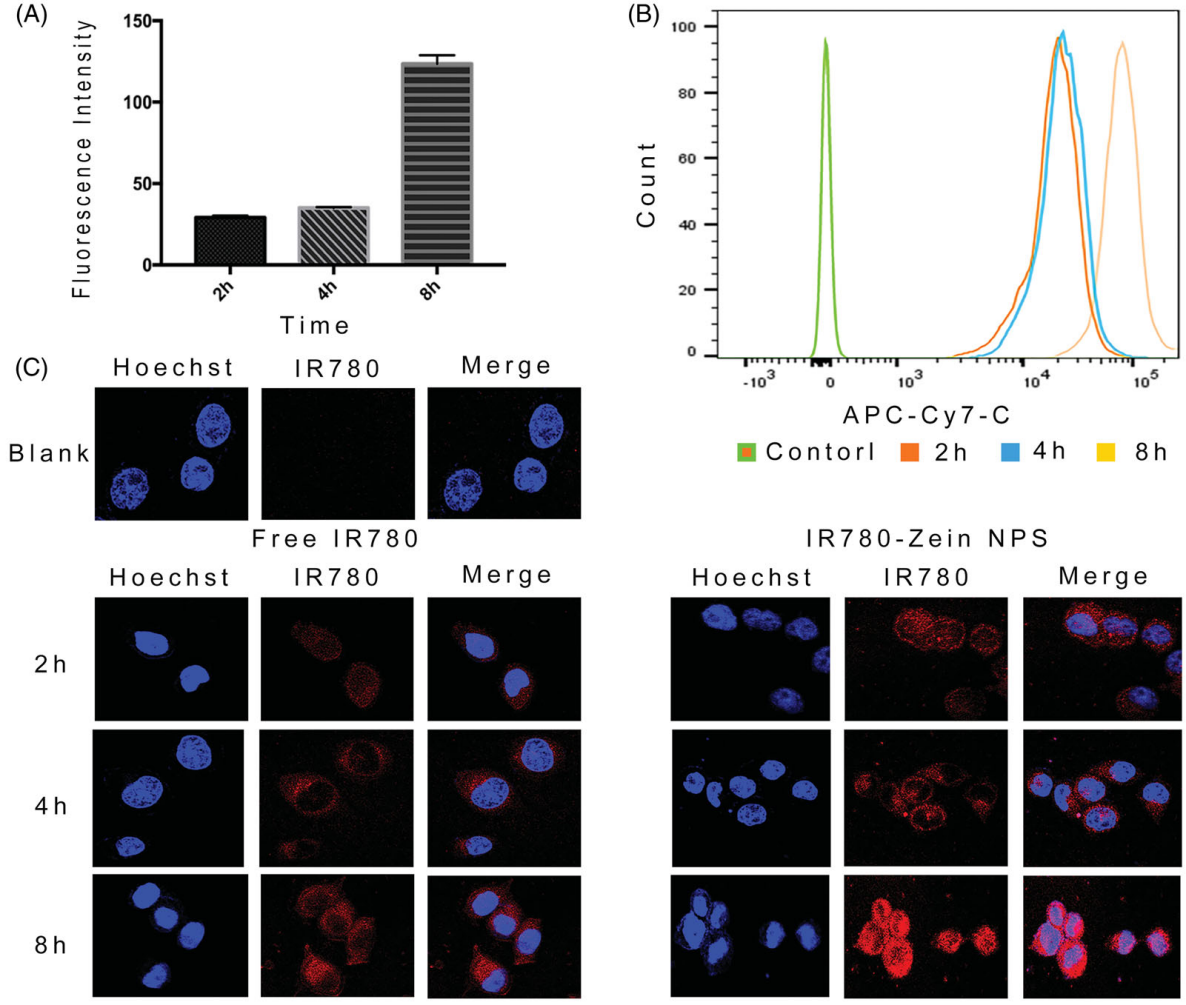
Flow cytometry and confocal microscopy images. (A and B) flow cytometry data of A549 cells incubated with IR-780-loaded ZNPs by recording IR-780 fluorescence. (C) Confocal fluorescence images of A549 cells incubated with IR-780-loaded ZNPs for 2, 4, 8** **h relative to control untreated cells. Blue and red colors represented hoechst-stained cell nuclei and IR-780 fluorescence, respectively. Error bars show SD. **p*** ***<**0**.01 (*n*** **=** **3).

The cellular uptake of ZNPs were assessed by flow cytometry and confocal laser scanning microscopy and the results were showed in Figure 3. As expected, both flow cytometry and confocal imaging results revealed strong fluorescence observed on A549 cells incubated with IR-780-loaded ZNPs, while cells treated with free IR-780 showed much weaker fluorescence (Figure 3(C)). The results indicated that the uptake of ZNPs by A549 tumor cells gradually accumulated over time. When entered the tumor cells (Figure 3(A,B)), it can increase the distribution in A549 cells nucleus (Figure 3(C)). Therefore, ZNPs not only increased the uptake of cells, but also increase the distribution in the nucleus after entered the A549 cells.

### Biodistribution study

**Figure 4.**
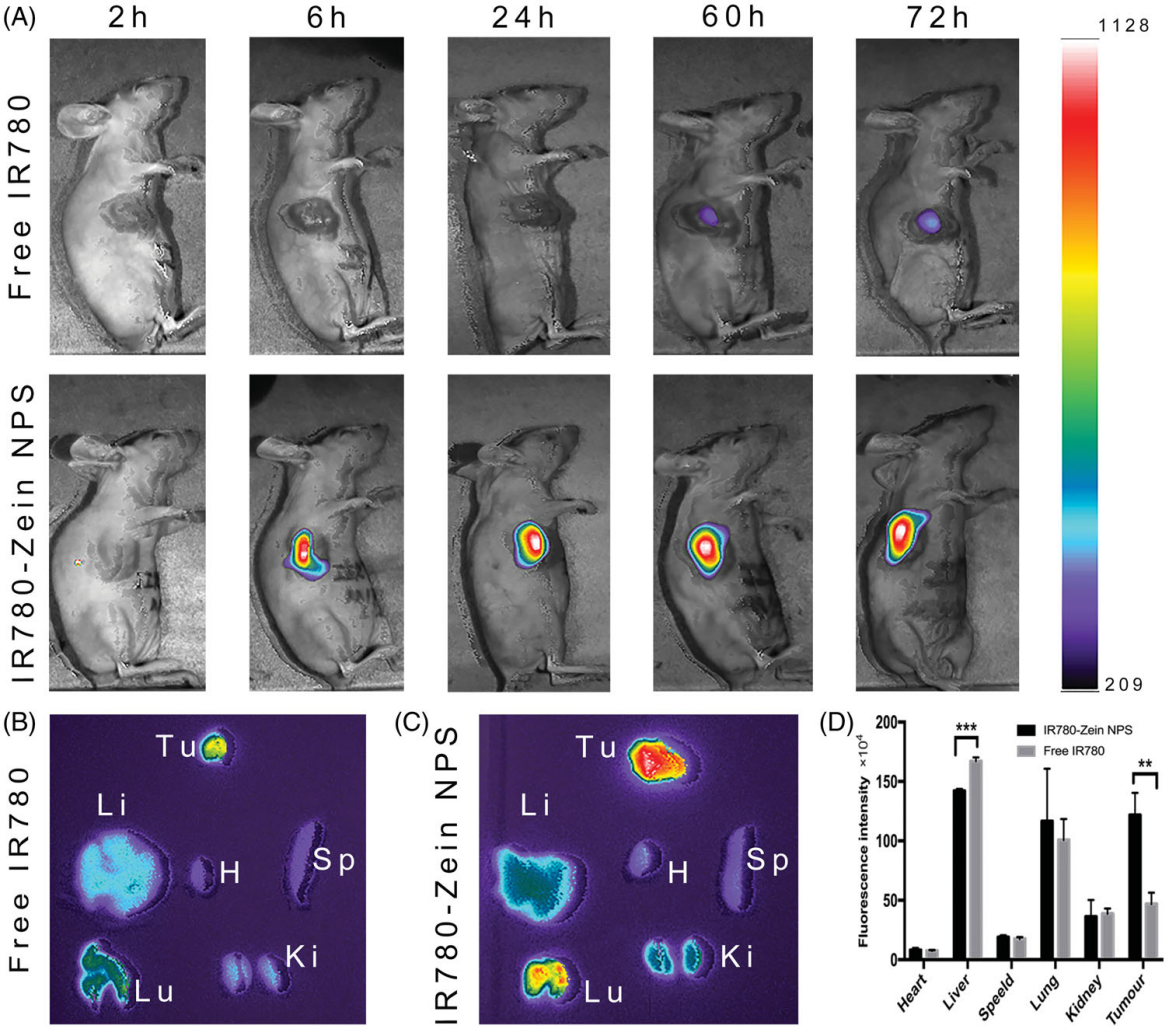
In vivo and ex vivo fluorescence imaging. (A) In vivo fluorescence images of A549 tumor-bearing nude mice taken at different time points post i.v. injection of free IR-780 (20** **µg) and IR-780-loaded ZNPs (20** **µg IR780 equiv.). Ex vivo fluorescence images of major organs and tumor dissected from mice injected with free IR-780 (B) and IR-780-loaded ZNPs (C) at 60** **h. Tu, H, Li, Sp, Lu and Ki stand for tumor, heart, liver, spleen, lung and kidney, respectively. (D) Semiquantitative relative biodistribution of free IR-780 and IR-780-loaded ZNPs in various organs as determined by the fluorescence intensities measured software. **p*** ***<**0**.01, ***p*** ***<**0**.001 (*n*** **=** **3).

The biodistribution study of the ZNPs in tumor-bearing mice results showed that the IR-780 fluorescent signal does not appear in the tumor at early time points. At later time points (after 6** **h and later), ZNPs showed obviously higher tumor accumulation than free IR-780, suggesting the specific tumor targeting ability of ZNPs (Figure 4(A)). Ex vivo imaging at 60** **h post injection also revealed that IR-780 fluorescence intensities of IR-780-loaded ZNPs group were 2.5 times higher than free IR-780 group (Figure 4(B–D)). Notably, the IR-780 signals of IR-780-loaded ZNPs group appeared to be significantly reduced accumulation in the liver and increased accumulation in the lungs.

### *In vivo* antitumor efficacy

**Figure 5.**
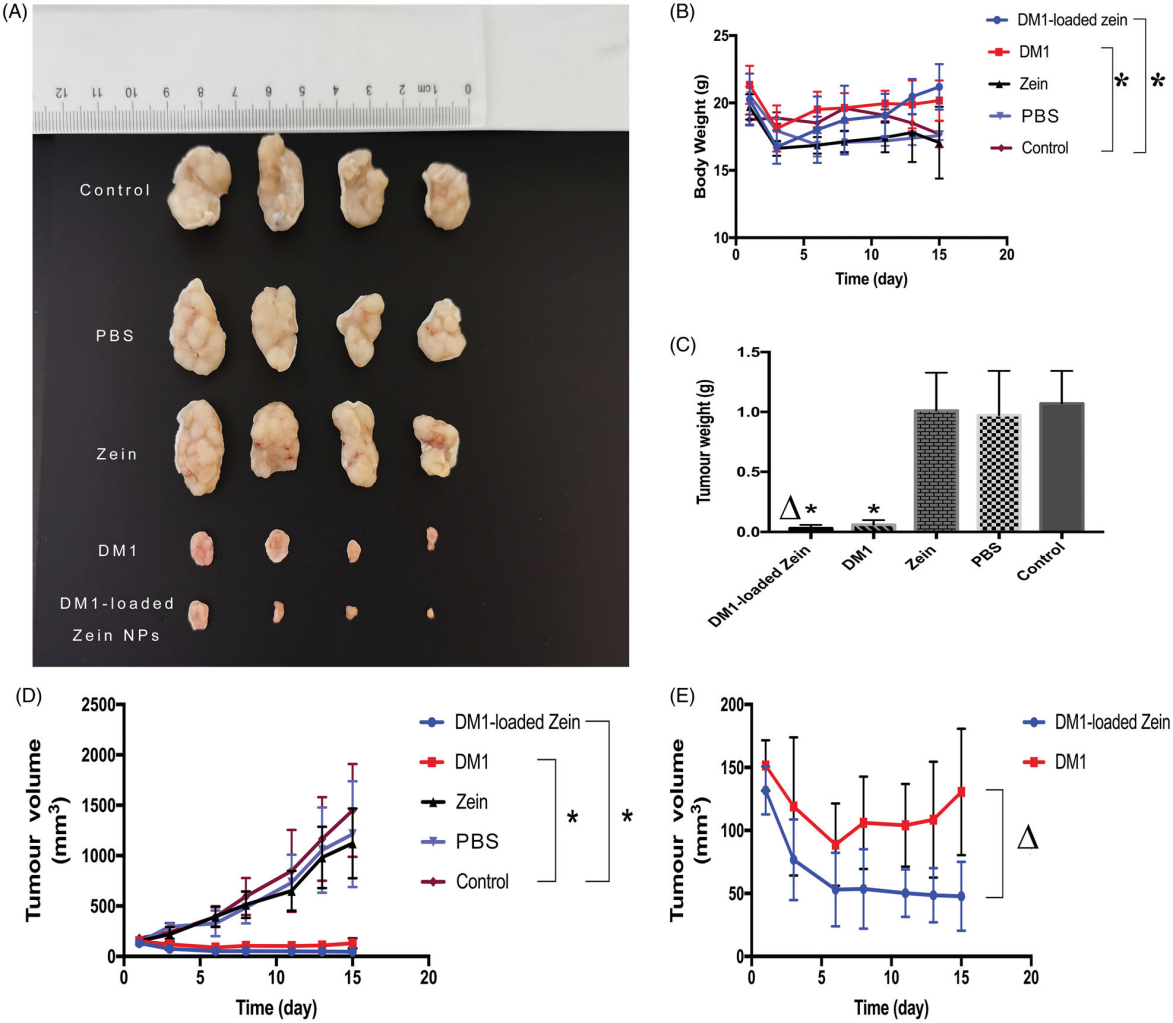
In vivo antitumor efficacy. (A) Photographs of typical tumor blocks collected from different treatment groups. (B) Change of mice body weights following different treatments. (C) Tumor weights collected from different treatment groups. (D) Tumor volumes changes of A549 tumor bearing nude mice treated with DM1-loaded ZNPs, free DM1, Zein, PBS and control, respectively. The results were presented as mean ± standard deviation (*n*** **=** **4). *p** **<** 0**.05, **p** **<** 0**.01 compared to control. (E) The tumor volumes of DM1-loaded ZNPs, free DM1, *Δp**** ***<** 0**.05 compared to free DM1.

**Figure 6.**
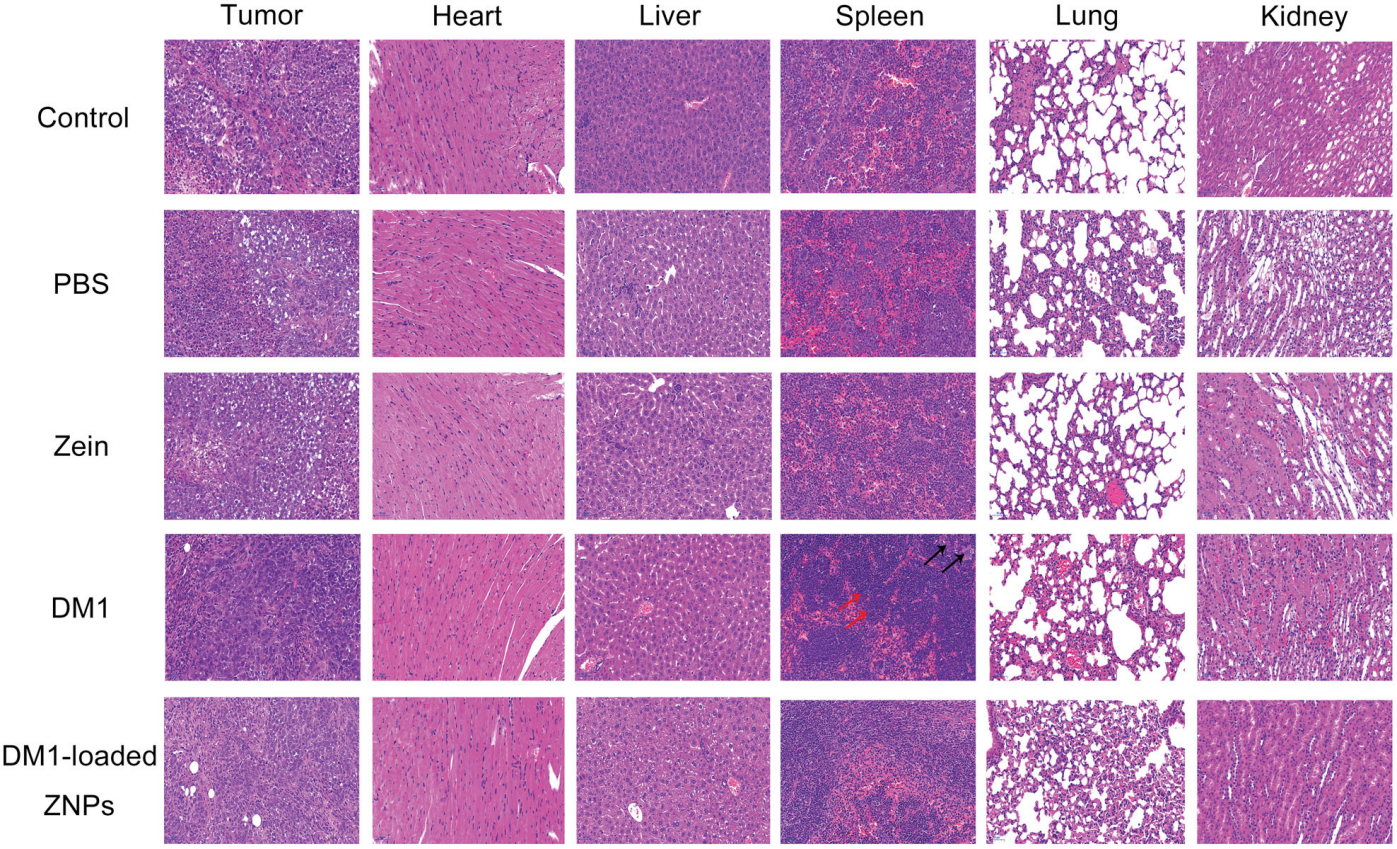
H&E staining of mice major organs from different groups. The spleen tissue of DM1 indicate the obvious reduced of splenic white pulp and lymph, leading to spleen damage. A large number of extramedullary hematopoietic cells (red arrows) in the red pulp, and multinuclear giant cell proliferation (black arrows), suggesting an inflammation in the spleen. The images were acquired using Pannoramic MIDI (3DHISTECH, EU) at 20× objective.

To evaluate *in vivo* anticancer effects, DM1-loaded ZNPs was administrated into A549 tumor-bearing nude mice at 0.8** **mg DM1 equiv./kg. As expected, control group, PBS and zein mice showed aggressive tumor growth. Both Free DM1 and DM1-loaded ZNPs displayed considerable tumor growth inhibition at a dose of 0.8** **mg/kg (Figure 5(D)). Moreover, DM1-loaded ZNPs displayed better tumor suppression significantly than free DM1 under the same conditions. After administration of the drug, the tumor tissue decreased sharply in the first five days. Thereafter, the tumor tissue of the DM1-loaded ZNPs group continued to decrease slowly, while the tumor tissue of the free DM1 group began to grow (Figure 5(E)). Interestingly, DM1-loaded ZNPs group tumor progression was completely suppressed at 0.8** **mg DM1 equiv./kg (Figure 5(A)). The weight of tumor-bearing mice showed a gradually decline and the body weight of the mice after treatment was significantly better than the control group (Figure 5(B)). On day 15, mice of each group were sacrificed and tumors were collected, weighed and photographed. The images of tumors showed that mice treated with 0.8** **mg DM1 equiv./kg DM1-loaded ZNPs had the smallest tumor size (Figure 5(A)), supporting that DM1-loaded ZNPs leads to the most efficient tumor growth inhibition. The weights of tumor blocks indicate that DM1-loaded ZNPs, DM1, zein and PBS yielded tumor inhibition rate (TIR) of 97.3%, 92.7%, 5.50%, and 9.10%, respectively. For the reason that DM1-loaded ZNPs yielded better tumor inhibition than DM1, zein and PBS (Figure 5(C)). H&E staining (Figure 6) displayed that DM1-loaded ZNPs at 0.8** **mg DM1 equiv./kg did not cause significant damage to the main organs while free DM1 induced obvious spleen damage, in line with the report that DM1 had a high cytotoxic potency in spleen (Labib, 2018). All the above results demonstrate that DM1-loaded ZNPs has improved toleration, better selectivity and enhanced treatment of A549 lung cancer. The superb drug loading, easy fabrication and quick cellular uptake renders DM1-loaded ZNPs a potentially drug to NSCLC.

## Discussion

Zein is a natural protein of plant origin have been applied extensively in controlled drug and biomedical delivery systems owing to its safety and biocompatibility (Chen et al., 2015; Labib, 2018). Here we used zein designed to become targeted drug cargo. In this case, we demonstrated that ZNPs accumulate in the tumor site of tumor-bearing mice. In the cytotoxicity experiment, we found that DM1-loaded ZNPs have a stronger inhibitory effect on A549 cells than free DM1. By flow cytometry and laser confocal experiments, we found that ZNPs can increase cellular uptake. In tumor-bearing mice, DM1-loaded ZNPs showed significant antitumor effects compared to free DM1 and we did not observe any measurable toxicity following injection of DM1-loaded ZNPs.

In cell experiments, first, we investigated the inhibitory effects of free DM1, ZNPs, and DM1-loaded ZNPs on A549 cells at different concentrations. The experimental results show that ZNPs do not have the effect of inhibiting tumor cell proliferation, so the inhibitory effect of DM1-loaded ZNPs on tumors originates from DM1. Interestingly, DM1-loaded ZNPs showed a stronger inhibitory effect on A549 cell proliferation than free DM1 at low dose, which should be related to that DM1-loaded ZNPs were more easily taken up by A549 cells. We observed stronger fluorescence signals in tumor cells treated with ZNPs by flow cytometry and confocal laser scanning microscopy, demonstrating that ZNPs increase cellular uptake compared to free drugs. Moreover, there are also many reports that ZNPs can increase cell uptake (Zhang et al., 2016). Hashem et al. and Jayan et al. encapsulated resveratrol into zein nanoparticles, which improved mucoadhesive properties and tissue permeability, thereby increasing oral bioavailability (Hashem et al., 2015; Jayan et al., 2019). Another study showed that TPGS-coated zein nanoparticles significantly increased cellular uptake and membrane permeation (Zou & Gu, 2013). This absorption enhancement mechanism may be involved in the transcytosis of the particles. The uptake of cells depends on the charge, particle size and other surface properties of the nanoparticles (Iversen et al., 2011). Positively charged nanoparticles are more easily bound to the negatively charged cell surface and then combined with anionic proteoglycans, or receptors (if ligands are involved like transferrin or folate etc.) on the cell surface (Harush-Frenkel et al., 2007). After attachment to the plasma membrane, enter the cells by means of clathrin-mediated endocytosis, caveolaemediated endocytosis and macropinocytosis (Bus et al., 2018). Moreover, nanoparticles can also be mediated via endocytosis or vesicles across cell membranes without the need for any specific receptors, suggesting that nanoparticle traverse the cell membranes can be driven by general physicochemical interactions (Contini et al., 2018). Studies by Dong et al. have further shown that the endocytosis pathway of zein nanoparticles is not a caveolin-mediated or clathrin-mediated pathway, but macropinocytosis (Dong et al., 2016). Therefore, we suspected that DM1-loaded ZNPs enter cells by an endocytosis pathway, which might be internalized more quickly than free DM1 via active transport. But, this enhancement effect was saturated after the nanoparticles reach a certain number (Zou & Gu, 2013; Pang et al., 2018). So that, the cytotoxicity of the DM1-loaded ZNPs is stronger than that of the free DM1 at low doses, and their cytotoxicity tends to be uniform with increasing dose. At present, whether ZNPs can enhance cell uptake through mechanisms other than macropinocytosis still needs to be verified by further experimental exploration, more studies are required to clarify the related mechanisms and key factors which affect cellular uptake of the ZNPs.

In biodistribution experiments, compared with free IR-780, the signal of IR-780-loaded ZNPs at tumor sites increased significantly, indicating that excluding the distribution of IR-780 itself at tumor sites, ZNPs can effectively accumulate at tumor sites, which we believe is related to the enhanced permeability and retention (EPR) effect caused by the size of zein particles. The endothelium of blood vessels of tumor is more easily penetrated (Torchilin, 2011). Under hypoxia, rapidly growing tumor tissues recruit new vessels or engulf existing blood vessels to form leaky vessels. These newly formed leaky vessels allow the passage of macromolecular substances more than 40 KDa. In addition, the lack of lymphatic drainage in tumors contributes to the retention of nanoparticles, while in the same case small molecule drugs are rapidly washed out of the tumor tissue (Attia et al., 2019). Particle size is an important factor affecting the EPR effect in tumors, limited by the tumor fenestrations in tumors vessels (200–800** **nm) ( Chono et al., 2007; Torchilin, 2011). On the other hand, when the particle size is less than 6** **nm, it is excreted by the kidney and more than 500** **nm passed through the reticuloendothelial system (RES). Therefore, in order to achieve tumor targeting, nanoparticles need to have a suitable size, which is 20–200** **nm (Kobayashi et al., 2014). We have also found that ZNPs significantly reduced the distribution in the liver and increased the distribution in the lung compared to free small molecule fluorescent developers. The liver was the main organ for the metabolic clearance of most drugs and exogenous substances. Phagocytic Kupffer cells and hepatocyte are the two main pathways for liver clearance. Particles larger than 200** **nm were cleared by Kupffer cells and particles 100–200** **nm were passed through endothelium of hepatic sinusoid and then enter the liver cells (Braet & Wisse, 2002). Airways and alveolar macrophages (AMs) are lung defense systems that selectively phagocytose particles larger than 100** **nm and NPs tend to agglomerate due to interparticle Ions in the aqueous airways where NPs meet AMs will compress the electrical double layer on the NPs surface, leading to further agglomeration (Wang et al., 2013). In this study, ZNPs have suitable size (about 110** **nm) can accumulated in the tumor site by the EPR effect, but the liver and lung can capture ZNPs by the endothelium of hepatic sinusoid and the alveolar macrophages, respectively. Moreover, ZNPs with high cationic charge density show aggregation in microvasculature of some organs such as liver, especially the lung (Saadat et al., 2019). As a result, zein has more distribution in the liver and lungs.

In vivo antitumor activity experiments, DM1-loaded ZNPs showed strong antitumor activity, which not only inhibited tumor growth, but also gradually reduced tumor tissue. According to the tumor volume curve, the tumor tissue volume decreased sharply in the first five days after administration of free DM1 or DM1-loaded ZNPs. After that, the tumor tissue of DM1-loaded ZNPs group continued to decrease slowly, while the tumor tissue of free DM1 group began to grow. This suggests that DM1-loaded ZNPs can inhibit tumor tissue growth more slowly, which may be related to the sustained release and long circulation characteristics of zein nanoparticles. That is, DM1 can be circulated in the blood for a longer period of time after being encapsulated by zein to form nanoparticles, and it has been reported in the literature that zein can continuously release the drug for more than 20** **days after the drug is wrapped (Luo & Wang, 2014). Nanoparticles can accumulate in the tumor site by the EPR effect, but due to the characteristics of the tumor tissue, most of the nanoparticles only stay at the periphery of the tumor tissue, and it is difficult to penetrate to the core of the tumor, so that the effect on the tumor tissue is limited. Thus, a drug delivery system can be slowly release, long circulation *in vivo* was very important, and zein nano drug delivery system was selected. According to the results of our cell experiments, the IC50 of DM1-loaded ZNPs was 0.01237** **ng/mL, which was lower than that reported in the literature, demonstrating that DM1-loaded ZNPs are more sensitive to A549 cells ( Reddy et al., 2007; Altai et al., 2018). We speculate that in this study, DM1-loaded ZNPs is sensitive to A549 cells and has strong antitumor growth effect. On the other hand, DM1-loaded ZNPs has the characteristics of sustained release and long circulation, which can continuously deliver DM1 to the tumor. Therefore, it exhibits excellent antitumor activity in vivo. However, whether A549 tumor cells have a specific receptor-mediated targeting of ZNPs still requires further research.

In this study, DM1-loaded ZNPs were prepared by phase separation method and the preparation conditions were optimized by BBD experimental design. It has the advantages of simple preparation method, low cost and high drug-binding et al., and can be used for large scale production. Cell and animal experiments have shown that DM1-loaded ZNPs exhibits strong anti-A549 tumor cell activity *in vitro* and in vivo. In animal experiments, the tumor inhibition rate of DM1-loaded ZNPs was 97.3%. This is related to the ZNPs enhance cellular uptake and accumulate in tumor by the EPR effect. Therefore, DM1-loaded ZNPs can be used as a promising treatment for non-small cell lung cancer. However, whether there is a special receptor for ZNPs in A549 cells, thereby increasing the cellular uptake need further research.
